# Monkeypox infection and pregnancy in lower and middle-income countries: Precautions & recommendations

**DOI:** 10.61622/rbgo/2024rbgo54

**Published:** 2024-06-27

**Authors:** Omar Abu-Azzam, Mohammad Abu-Jeyyab, Mohammad Daradkeh, Sadeen Zein Eldeen, Saja Zuaiter, Mohammad Al-Mseadeen, Amer Sindiani, Eman Alshdaifat

**Affiliations:** 1 Department of Obstetrics and Gynecology School of Medicine Mutah University Al-Karak Jordan Department of Obstetrics and Gynecology, School of Medicine, Mutah University, Al-Karak, Jordan.; 2 School of Medicine Mutah University Al-Karak Jordan School of Medicine, Mutah University, Al-Karak, Jordan.; 3 Department of Obstetrics and Gynecology Faculty of Medicine Jordan University of Science and Technology Irbid Jordan Department of Obstetrics and Gynecology, Faculty of Medicine, Jordan University of Science and Technology, Irbid, Jordan.; 4 Department of Obstetrics and Gynecology Faculty of Medicine Yarmouk University Irbid Jordan Department of Obstetrics and Gynecology, Faculty of Medicine, Yarmouk University, Irbid, Jordan.

**Keywords:** Monkeypox, Orthopoxvirus, Pregnancy complications, infectious, Perinatal care, Epidemiology

## Abstract

Monkeypox (MPX), an orthopoxviral disease endemic in Africa, is now a public health emergency of international concern (PHEIC) as declared by the World Health Organization in July 2023. Although it is generally mild, the overall case fatality rate was reported to be 3%, and the basic reproduction number (R0) is > 1 in men who have sex with men (MSM, i.e., Portugal (1.4), the United Kingdom (1.6), and Spain (1.8)). However, R0 is < 1 in other settings. In concordance with the smallpox virus, it is also expected to increase the risk of adverse outcomes for both the mother and the fetus. The outcomes of the disease in an immunocompromised state of pregnancy are scary, showing high mortality and morbidity of both mother and fetus, with up to a 75% risk of fetal side effects and a 25% risk of severe maternal diseases. Therefore, it warrants timely diagnosis and intervention. The reverse transcription polymerase chain reaction (RT PCR) test is the standard approach to diagnosis. We summarized the recent findings of MPX on pregnancy, and the associated risk factors. We also give recommendations for active fetal surveillance, perinatal care, and good reporting to improve outcomes. The available vaccines have shown promise for primary disease prevention.

## Introduction

Monkeypox (MPX) is a viral zoonosis caused by the monkeypox virus (MPXV), which is an Orthopoxvirus closely related to the variola virus causing smallpox.^(1)^ Although it has been endemic to some African countries, MPX has spread its roots to non-endemic countries.^(2)^ As of December 24, 2022, the Centers for Disease Control and Prevention (CDC) showed a total of 83,424 MPX-confirmed cases, including children, in 86 countries worldwide.^(3)^ The first documented case of MPX in a human being occurred in 1970. Various nations of central and western Africa have previously witnessed human-to-human transmission of MPX before the 2022 multi-country outbreak.^(4)^ Due to this sudden increase in MPX-confirmed cases and change in transmission modes, the World Health Organization (WHO) declared MPX a public health emergency of international concern (PHEIC) as of July 23^rd^, 2022.^(5)^ The MPX clade (II), having a lower-case fatality rate (3.6%) in comparison with the clade (I) (10.6%), has been attributed to the current multi-country outbreak.^(6)^ MPX severity has been seen to be more among vulnerable groups of individuals, which indicates increased risk for children and pregnant females, though the data is limited.^(7)^ Thus, in this study, we aim to review the recent literature on MPX in pregnancy and the associated risk.

### Routes of transmission

MPX can spread to people by bites or touching the infected blood, flesh, body fluids, or cutaneous/mucosal lesions of the animal such as Rope, sun squirrels, and African dormice. Sustained human-to-human transmission has been documented, typically involving prolonged, intimate contact or significant droplets of breathed air.^(8)^ It is also possible for the virus to be spread through sexual contact (particularly among homosexuals, bisexuals, and men who have sex with men (MSM));^(9)^however, it is not known whether this happens when there is genuine sexual contact or just when there is close touch during intercourse. Despite the scarcity of available information, human-to-human transmission risk appears to be modest; however, the highest risk group of severe MPX forms in infants and young children. According to Thornhill et al.,^(10)^ sexual close contact was the likely mode of monkeypox viral transmission in 95% of the cases.

Further studies are still needed to confirm whether or not infected mothers transmit the virus to their unborn children. Furthermore, research is still conflicting regarding the way of MPX maternal-fetal transmission (e.g., nursing droplet transmission, close contact with skin lesions of the mother); however, vertical transmission of MPXV between the mother and her newborn has been confirmed.^(11)^

MPX is endemic to tropical rainforest areas of central and west Africa and rarely presents an outbreak in other countries. The status of smallpox vaccination and the expected reservoirs support the occurrence in tropical regions of lower and middle-income countries (LMIC).^(12)^ The data for LMIC and high-income countries is limited and will add as the literature grows. Still, more severe outcomes might occur for people infected with LMIC, especially immunocompromised and pregnant females.^(13,14)^

Various reports of maternal and fetal outcomes following an MPX infection during pregnancy have underlined to 75% risk of side fetal effects and a 25% risk of severe maternal diseases.^(15)^ In the Democratic Republic of Congo report, three women reported adverse events; two of them had a spontaneous miscarriage in the first trimester accompanied by a moderate-to-severe form of MPX; however, the third one had intrauterine fetal demise.^(16)^ Several serological, histological, and virological evidence confirm that fetal death was because of MPXV vertical transmission.^(15)^ The overall risk of first-trimester miscarriage has been reported as 25–30%.^(14)^ A report also shows an increased risk of premature delivery and poor fetal outcomes due to malnutrition, however, it was not laboratory-confirmed.^(14)^ The examined fetus has shown characteristics of skin lesions, hepatomegaly, and peritoneal effusion (hydrops fetalis), which can be attributed to increased vascular permeability, MPXV-induced cellular injury, and placental cytokine response.^(17)^

### Manifestations

In contrast to smallpox, the initial signs of monkeypox include fever, headache, myalgia, tiredness, and lymphadenopathy. After a 1–2-day period, the mouth starts to have mucosal lesions, followed by skin lesions that are more centrally located on the face and extremities (including the palms and soles). A small number of lesions may appear on the skin, or there may be hundreds. The rash may extend to other parts of the body, or it may not. Over the next 2–4 weeks, the lesions progress through macular, papular, vesicular, and pustular stages, each lasting 1–2 days. Changes in lesions occur simultaneously and are characterized by a hard, deeply rooted lesion from 2 mm to 10 mm in size. Pustules on lesions last around 5–7 days before forming crusts. Typically, the illness clears up about three to four weeks following the commencement of symptoms, during which time crusts develop and desquamate. Once all crusts have fallen off, a patient is no longer contagious.^(18)^

Chart 1 summarizes rash progression across time.

**Chart 1:** Monkeypox rash progression

Lesion progression	Timing
Enanthem	Mucosal lesions start developing 1-2 days after prodromal symptoms
Stage 1: Macules	The rash starts as flat red spots that last 1-2 days
Stage 2: Papules	The rash starts as flat red spots that last 1-2 days
Stage 3: Vesicles	small, clear, fluid-filled blisters < 10 mm in diameter lasting 1-2 days
Stage 4: Pustules	Umbilicated lesions filled up with pus lasting for 5-7 days
Stage 5: Scabs	Pustules crust over and scabs will remain for about 1 week before eventually falling off

It may be difficult to distinguish the source of fever during pregnancy from other illnesses such as intraamniotic infection (chorioamnionitis) until the rash occurs; therefore, it’s important to rule out those possibilities. When pregnant women have a fever, it’s crucial to rule out other possible causes, such as an intraamniotic infection (chorioamnionitis). Pregnant women, at risk for monkeypox virus infection, and who also have a rash should be checked for other dermatoses of pregnancy, such as the polymorphic eruption of pregnancy, which is also known as pregnancy pruritic urticarial plaques and papules. Moreover, MPX-infected lesions and rashes may be misdiagnosed with those of other common illnesses like varicella zoster or sexually transmitted illnesses (STIs) and thus should be carefully evaluated for a characteristic monkeypox rash, and diagnostic testing should be considered, especially if the patient has epidemiologic risk factors for monkeypox virus infection.

Secondary infections, bronchopneumonia, sepsis, encephalitis, and cornea infection leading to vision loss have all been documented as consequences of monkeypox.^(12)^ Previous studies indicated that between 1-10% of MPX-related fatalities occurred. However, current case-fatality rates cannot be estimated due to a lack of reliable data on the actual number of cases and deaths.^(19)^

**Figure 1:**
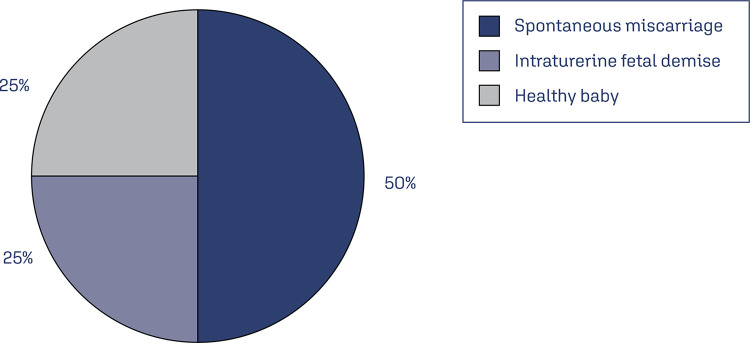
A summary of Mbala et al.(17) study of birth complications by monkeypox

Regarding maternal and fetal complications, a pregnant woman infected at the 24-week gestation had a preterm baby 6 weeks after. This infant died of malnutrition 6 weeks after developing a monkeypox-like rash. In another study summarized in figure 1,^(17)^ the information relies on case reports, and the most extensive set of monkeypox cases recorded in a single series encompasses a total of 222 cases. Four pregnant women were among 222 symptomatic Democratic Republic of the Congo (DRC) patients hospitalized with monkeypox between 2007 and 2011. Three of the four women had stillbirths, and one had a healthy baby. Three women have reported adverse events; two had a spontaneous miscarriage in the first trimester accompanied by a moderate-to-severe form of MPX; however, tissue testing was not done. Further, the third one had intrauterine fetal demise at 18 weeks.^(16)^

### Diagnosis and case detection

If health workers suspect monkeypox, they should collect a specimen to submit to a reputable lab for analysis. To confirm an MPX case, a skin lesion sample, and a PCR lab test are required. Package and ship specimens following applicable local, state, federal, and international laws.^(20)^ Polymerase chain reaction (PCR) is the most precise and sensitive scientific test. Vesicles, pustules, and dry crusts on the skin may be used to diagnose monkeypox. Sometimes a biopsy is the only option. There must be a steady supply of cold, dry air around lesion samples (no viral transit medium). Normal PCR blood tests should not be performed on patients because of the short time of viremia (1-2 days) concerning specimen collection after symptoms begin.^(21,22)^ Due to the cross-reactivity of Orthopoxviruses, MPX cannot be detected using antigen and antibody detection methods. When funds are scarce, serology and antigen detection are not recommended. False positive results may occur if the individual was vaccinated with a vaccinia-based vaccination either recently or in the past (e.g., before smallpox eradication or more recently due to heightened risk, such as Orthopoxviruses laboratory workers).^(23)^ Patient information, including age, current state (stage of rash), and the date of fever onset and rash onset, must be provided with specimens to interpret test results. The clinical differential diagnosis includes varicella, measles, scabies, syphilis, and allergy to medications.^(24)^Chart 2 further portrays the key clinical characteristics for differentiating between monkeypox, smallpox, and varicella zoster infection.

**Chart 2:** Key clinical characteristics to differentiate between Smallpox, Monkeypox, and Varicella

Characteristics	Monkeypox	Smallpox	Chickenpox
Causative virus	Monkeypox virus	Variola virus	Varicella zoster virus
Fever	1-3 days before the rash between 38.5°C and 40.5°C	2-4 days before the rash often >40°C	1-2 days before the rash up to 38.8°C
Initial site and lesion appearance	Face Hard and deep, well-circumscribed, umbilicated	Tongue and mouth Hard and deep, well circumscribed, umbilicated	Chest, back, and face Superficial, irregular borders, “dew drop on a rose petal”
Lesion progression	one stage of development on the body; slow progression with each stage lasting 1–2 days	one stage of development on the body; slow progression with each stage lasting 1–2 days	multiple stages of development in the body; fast progression
Lymphadenopathy	Yes	No	No
Headache	Yes	Yes	Yes
Duration of illness	2-4 weeks	Up to 5 weeks	4-7 days

### Management

The prevention of MPX transmission in pregnant women is similar to non-pregnant women. Pregnant women should avoid close contact with either suspected or confirmed MPX cases.^(19)^ Smallpox vaccines can protect against MPX infection with an efficacy of 85%. Although vaccination against MPX should be considered in pregnant women, evidence is limited in this area, calling for more research in this regard. Minimally replicating and non-replicating smallpox vaccines (i.e., JYNNEOS (MVA-BN) and LC16) are considered for pre-or post-exposure prophylaxis in pregnant and breastfeeding women. ACAM2000, another smallpox vaccine, has limited evidence regarding its use during pregnancy and breastfeeding. Thus, it should be avoided in pregnant women until enough evidence is provided. Additionally, several side effects have been reported with ACAM2000^(23)^ as shown in chart 3.

**Chart 3:** MPX vaccines and possible use and reported adverse events during pregnancy/breastfeeding

Variables	Vaccine		
	ACAM2000	LC16	JYNNEOS
Pregnancy	N/A	cautiously	Yes
Breastfeeding	N/A	cautiously	Yes
Side effects			
lymph node pain	Yes	N/A	N/A
lymphadenopathy	Yes	Yes	N/A
Nausea	Yes	N/A	Yes
Diarrhea	Yes	N/A	N/A
Headache	Yes	N/A	Yes
Fatigue	Yes	Yes	Yes
Constipation	Yes	N/A	N/A
Vomiting	Yes	N/A	N/A
Inadvertent inoculation	Yes	N/A	N/A
Dyspnea	Yes	N/A	N/A
The presence of a “take” following vaccination	Yes	N/A	N/A
Allergic reactions	Yes	Yes	N/A
Injection site purities	Yes	Yes	Yes
Injection site pain	Yes	Yes	Yes

N/A - information not available

JYNNEOS, a non-replicating virus vaccine, can be used during pregnancy due to low concern regarding its side effects.^(19)^ Still, JYNNEOS in pregnancy should only be used when the benefits outweigh the potential risk of developing adverse events.^(14)^ The European Medicines Agency has reported that the JYNNEOS vaccine could be effectively used during pregnancy with no major adverse events, according to a study conducted on 300 pregnant women.^(19)^ Furthermore, the rates of preterm birth, pregnancy loss, and birth defects were similar among pregnant women receiving smallpox vaccination compared with non-pregnant women, according to a nationwide registry-based investigation in the USA.^(19)^ Additionally, it has been highlighted that no cases of the vaccinia virus were reported.

It has been reported that smallpox vaccines contraindicated during pregnancy are safe during breastfeeding, i.e., JYNNEOS; However, if it passes in the milk, it does not have potential risks due to the defective replication in humans. Accordingly, vaccinating pregnant women should be considered, especially at a high risk of exposure. Additionally, balancing the maternal-fetal risks and benefits is recommended.^(14)^

No specific treatment has been licensed for MPX, but some antiviral drugs (i.e., tecovirimat (TPOXX), Brincidofovir, and cidofovir) can be considered for the management of this infection.^(19)^

U.S. Food and Drug Administration (FDA) prescribing information for tecovirimat reports that no embryotoxic and teratogenic effects have been detected in animal studies, and US CDC guidelines state that tecovirimat should be considered as the first-line antiviral for pregnant women, recently pregnant, or breastfeeding.^(25)^ On the other hand, Cidofovir is considered a category C drug in pregnancy secondary to proven teratogenic and embryotoxic effects, including skeletal and soft tissue abnormalities and small-for-gestational-age, according to the FDA, and based on evidence from animal studies.^(19)^

The administration of vaccinia immunoglobulin (VIG) via intravenous immunoglobulin therapy has also been reported to be effective in treating smallpox, making the USA-CDC criteria license its use in MPX cases during the 2003 MPX outbreak in the USA.^(19)^However, the efficacy and safety of this therapy in MPX have not been supported by sufficient evidence so far.^(26)^ Although evidence of the use of VIG in pregnancy is lacking, the safety of other immunoglobulins used in pregnancy is established.^(19)^

### Healthcare systems preparedness

**Figure 2:**
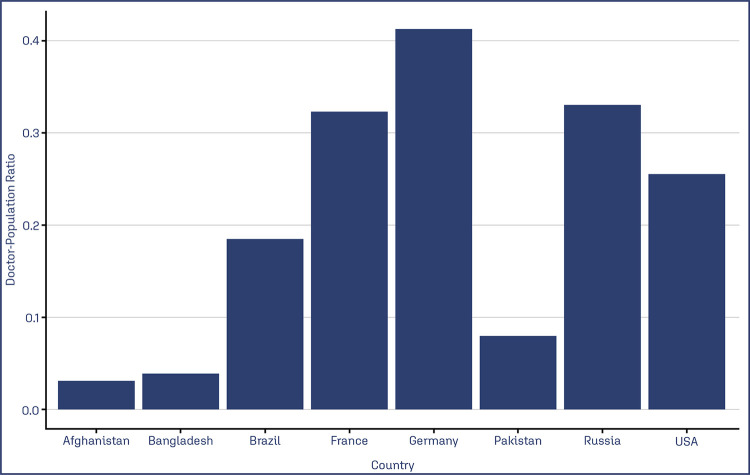
Health sectors economics for infectious health in low- and moderate-income countries

As a cornerstone concept, the lower the national income, the poorer the health care equity, and the fewer the protocoled services available. In LMIC countries, there commonly are no separate healthcare beds, ventilatory or cardio-respiratory support facilities, and a high provider-receptor ratio. According to number-based studies, these are some examples of the doctor population ratio in Low and middle-income countries: Bangladesh, 0.389:1,000; Pakistan, 0.806:1,000 and Afghanistan, 0.304:1,000, and Brazil, 1.852:1,000 compared to the USA, 2.554:1,000; Germany, 4.125:1,000; France, 3.227:1,000; Russia, 3.306:1,000 (Figure 2).^(27)^Consequently, this was reflected in the maternal-fetal units; maternity professionals attend 3.8 million births, and over 10% of them require treatment in a neonatal intensive care unit (NICU) annually.^(28)^

That effect was established even more during the COVID-19 pandemic. Many national health systems in LMICs are unable to afford pharmaceuticals like vaccines, diagnostic instruments, and antivirals due to economic instability and the toll COVID-19 took on healthcare systems. Such infectious disease outbreaks highlight the insufficiency and lacking technology in some LMICs that include but are not limited to paper mode registrations, electricity cut-offs, and poor medical materials supplements (i.e., personal protective equipment (PPEs), Oxygen supplements, and deficient medications needed in treating these patients). That is why it is imperative that international financing be made easily accessible to help LMI countries deal with the evolving situation and create a global extensive surveillance system to comprehend the continuously evolving epidemiology of this emergent sickness.^(29)^

### Recommendations to pregnant women

It is paramount to make the general public, especially pregnant women, aware of the MPX symptoms, transmission, prevention, and available antivirals and vaccines against MPX in LMIC. More importantly, the awareness of MPX-infected pregnant women should be enhanced regarding contact with their newborns and breastfeeding. Direct close contact with the newborns is not recommended until the mother becomes fully recovered by achieving the CDC criteria. However, if the patient insists on directly contacting her baby, some precautions should be considered (1) No direct or close contact. (2) The newborn should be entirely clothed, and the clothes should be replaced after contact. (3) The patient should wear gloves and well-fitting medical masks while contacting the baby to avoid the chances of viral transmission. MPX has not been proved or aborted to be transmitted through milk. Thus, and for the baby’s safety, breastfeeding should be avoided until the mother becomes fully recovered by achieving the CDC criteria (e.g., resolving all infected lesions, falling off all scabs, and forming a fresh layer of intact skin).^(23)^ Reducing the risk of newborn MPX infection appears fair in high-income countries like the UK. But in LMIC nations, the advantages of breastfeeding may offset the higher risk of newborn MPX infection, which might be difficult due to economic and nutritional difficulties. They might subsequently outweigh the risk of transmitting the infection.^(19)^

### Recommendations to governments and policymakers

Governments and policymakers in LMIC should take rapid and decisive actions to keep it under control. The moment has come to take a genuinely global approach to this disease, especially after declaring it as PHEIC by the WHO.^(5)^ They should learn from previous outbreaks (i.e., COVID-19) to halt the MPX outbreak before bursting out of control, especially with an outbreak with such an unexplained widescale spread. In less developed areas, it’s also critical to provide medical workers with necessary resources (i.e., PPE, antivirals, and vaccines). In addition, governments should increase funding for studies investigating the causes behind the sudden widescale outbreak of MPX and the change in transmission modes.^(30)^

### Recommendations to healthcare professionals

This re-emerging pathogen must be recognized and dealt with by healthcare workers. Morbidity among the medical staff would increase the load on the healthcare system during an outbreak, so doctors and nurses should take precautions to avoid MPX infection, like using PPEs that include a gown, gloves, eye protection, and a particulate respirator equipped with N95 filters or higher. Every medical team member must be aware of their specific responsibilities in this situation, which resembles the COVID-19 pandemic in some aspects.^(31)^ An interdisciplinary team of doctors, veterinarians, nurses, virologists, and, more importantly, public health professionals can quickly detect MPX cases in animals and humans, apply preventive measures, and commence immediate reporting of MPX infection, which is a safeguard against the severe progression of the recent MPX outbreak.^(32)^

### Recommendations to researchers

Regarding researchers, additional research is needed on the JYNNEOS vaccination. Following a two-dose JYNNEOS vaccination series, more investigations are needed to evaluate the length of protection; recommendations for the frequency of booster doses can be amended appropriately. If Orthopoxviruses exposures occur before peak immunogenicity is attained, a single dose JYNNEOS series should be considered. In addition, well-designed clinical trials are warranted to assess myopericarditis and other major side effects to examine how JYNNEOS interacts with the mRNA COVID-19 vaccinations. Identifying a correlate of protection after JYNNEOS vaccination might help establish the vaccine’s efficacy in pregnant women and give insight into the efficacy of a single dose of the JYNNEOS vaccine.^(33,34)^

### Recommendations for early preparedness of MPX in LMIC

#### Fetal surveillance

Methods of fetal surveillance are summarized in chart 4. Pregnant women infected with MPX are at an increased risk of transmitting the disease to their unborn children. The fetus and placenta should be checked often during acute illness. Screening during the first trimester is important to determine the viability. Fetal biometry, amniotic fluid volume measurement, and a thorough anatomy scan should be done 10–14 days apart in the second trimester of pregnancy.^(11,35)^Intermittent auscultation represents as a key approach to fetal surveillance during labor. This method involves the periodic assessment of the fetal heart rate (FHR) using a device, typically a handheld Doppler device or fetoscope, along with manual palpation to evaluate uterine contractions.^(36)^

**Chart 4:** Fetal surveillance

Gestational Age	Appropriate surveillance method
First trimester	Screening
Second trimester	Fetal biometry, amniotic fluid volume measurement, and a thorough anatomy scan

## Delivery considerations

Maternal MPX infection alone is not a reason to hasten delivery. Most cases, especially those of the MPX in West African lineage, causing the present multi-country outbreak, are not dangerous and self-limiting. A decision should be made about whether to deliver the baby should consider the mother’s health, the fetus’s condition, and the likelihood that the birth will benefit or harm the mother. A single steroid course for fetal development is unlikely to significantly influence maternal status. Neonatal neuroprotection with magnesium sulfate should be given per unit policy when planned preterm birth. Contact with open infected MPX lesions is known to spread the virus. A cesarean section (C-section) may not be necessary if the infant is infected at delivery, as the vertical transmission is likely. Giving birth to a child while suffering from genital sores increases the risk of infection in the newborn. As a result, and in the case of genital lesions presence, a C-section is paramount in cases when a newborn is at high risk of developing a serious MPX infection. C-section is fundamental even if the infected vaginal lesions are apparent in MPX-infected women or possible MPX infection incidence after discussing the unquantifiable risk of newborn MPX infection.^(11,17)^

## Neonatal care

A lack of evidence to guide newborn care for MPX-infected offspring is present. With PPE-clad caregivers, the baby should be born in an isolated room. Infants should be closely monitored for symptoms of sickness or MPX. A mother and child can be reunited if the infant tests positive. The WHO recommends that the newborn of a mother who has passed the monkeypox PCR threshold be isolated. The mother and kid should be reunited as soon as she receives two negative PCR findings.^(37,38)^

## Case reporting

Registries for pregnant women and other vulnerable populations should be established immediately in LMIC. Thus, future epidemics may be addressed more efficiently; every obstetrician should attempt to record instances. However, applying for such registries can be challenging in LMIC due to the limited resources and funding. Therefore, high-income countries and international organizations (i.e., WHO) should play a major role in this because if such outbreaks are not managed in an efficient and timely manner, the spread will not affect LMIC only but also high-income countries.
